# Influence of peer discussions on trust in recommendations for prevention of mother-to-child transmission (PMTCT) of HIV

**DOI:** 10.1371/journal.pone.0311109

**Published:** 2024-09-27

**Authors:** Rune Nathaniel Philemon, Innocent B. Mboya, Blandina T. Mmbaga, John Bartlett, Sia E. Msuya

**Affiliations:** 1 Department of Pediatrics and Child Health, Kilimanjaro Christian Medical University College, Moshi, Tanzania; 2 Department of Pediatrics and Child Health, Kilimanjaro Christian Medical Centre, Moshi, Tanzania; 3 Department of Epidemiology & Biostatistics, Institute of Public Health, Kilimanjaro Christian Medical University College, Moshi, Tanzania; 4 Department of Translational Medicine, Lund University, Malmo, Sweden; 5 Kilimanjaro Clinical Research Institute (KCRI), Moshi, Tanzania; 6 Duke Global Health Institute, Duke University, Durham, North Carolina, United States of America; University of Zimbabwe Faculty of Medicine: University of Zimbabwe College of Health Sciences, ZIMBABWE

## Abstract

**Background:**

Mothers attending prevention of mother-to-child transmission (PMTCT) of HIV clinics seem to lack knowledge on many aspects of PMTCT, among which is breastfeeding. Breastfeeding recommendations in PMTCT have changed several times over the years leaving some confused and doubtful of what is currently recommended. One method shown to help improve their knowledge and acceptance of PMTCT recommendations is the use of peer educators. We sought to determine if mothers engage in discussions with other mothers during clinics and how these engagements influence trust in PMTCT recommendations.

**Methods:**

We interviewed 524 mothers with children under two years enrolled in PMTCT clinics in Kilimanjaro, Tanzania. We selected 5 clinics with the highest numbers of PMTCT enrolment from each district in the region. In each clinic, over a one-month period, we recruited all mothers attending the PMTCT clinic. We collected information on their engagement in discussions regarding PMTCT during clinics and how they perceived the information from their peers in relation to that from healthcare providers.

**Results:**

Fifty-five percent of the mothers reported engaging in peer discussions. Of the 90 (17%) mothers who reported noticing a change in PMTCT recommendations, 33 (36.7%) reported trusting previous recommendations more. A greater proportion (52.9%) of mothers who engaged in peer discussions reported trusting the information from peers more than that from healthcare workers.

**Conclusions:**

Peers have a great influence on mothers, which is concerning when their knowledge shared is outdated. Harnessing their influence and training them on current recommendations might be key to improving adherence to PMTCT recommendations.

## Introduction

Since Human Immunodeficiency Virus (HIV) in children is mainly the result of maternal infection, prevention of mother-to-child transmission (PMTCT) remains a significant tool in the fight against HIV in children. Correct knowledge of PMTCT has been shown to impact success in PMTCT positively [1]. However, the knowledge mothers have regarding PMTCT recommendations is less than complete in many places [2–4]. Many key components in the PMTCT cascade elude mothers enrolled in PMTCT, even in components that the mothers are expected to perform unsupervised, e.g., breastfeeding [4]. The confusion about what is currently recommended in PMTCT does not end with the mothers alone. Even healthcare providers have been shown to lack understanding of PMTCT recommendations [5]. The changes in recommendations through the years as PMTCT has evolved have been blamed for the confusion [6]. Although these changes in recommendations are inevitable with the development of scientific evidence, there is a need to ensure that mothers are kept up to date regarding recommendations, as their understanding of them will influence their adherence and, subsequently, the success of PMTCT programs.

One of the best examples of the differing PMTCT recommendations has been breastfeeding. Early recommendations for PMTCT from the WHO recommended avoidance of breastfeeding for HIV-positive mothers where possible [7]. These recommendations have been updated across the years with growing evidence, and with it, national guidelines were also updated. Currently, Tanzania follows the updated option B+ recommendations stipulating that HIV-infected mothers breastfeed exclusively for the first 6 months and then stop breastfeeding altogether when the child is 12 to 13 months. The current guideline also recommends testing high-risk infants at birth and all infants at six weeks, as well as ARV prophylaxis for the first six weeks [8].

Although health education for mothers remains key to the success of breastfeeding in PMTCT, several studies have demonstrated that it is inadequate due to staff shortages and other challenges. Not all mothers are counseled on breastfeeding [4, 9]. Educating mothers on PMTCT cannot be left to healthcare providers alone as they are highly overworked, creating a communication gap between them and the patients [10]. Peer educators have been called in to cover this gap and have been highly successful in educating mothers on PMTCT, though not all countries and facilities are practicing this approach yet [1, 11].

Where formal peer educators have not been adopted, societal influencers such as other mothers, mothers-in-law, and grandmothers still strongly influence the opinion of many mothers [1]. The strong influence of these untrained peers in the community, coupled with the distrust that exists towards healthcare providers, means that mothers could potentially be getting PMTCT recommendations from their peers rather than well-informed sources [12]. This misinformation becomes dangerous when we consider the low level of knowledge that these peers might have [4]. We believe that the influence of peers might be a key to understanding why knowledge of PMTCT amongst mothers in the PMTCT program is low. Adequate knowledge among mothers in the PMTCT program and trusting the recommendations will help improve the utilization of PMTCT services and adherence to its recommendations [13]. The importance of adherence to PMTCT recommendations cannot be overemphasized if the PMTCT program is to be successful.

We sought to determine if mothers engage in discussions with other mothers (peers) during clinics and how these engagements influence trust in PMTCT recommendations. We also explored if they had noticed any changes in the breastfeeding recommendations given in PMTCT between their previous pregnancies and the pregnancy of the reference child for those who had previous PMTCT encounters. This paper describes how discussions with peers and healthcare workers influence mothers’ perceptions regarding PMTCT. We also explore whether mothers recognize the changes made to recommendations and whether or not this influences their perceptions. By identifying their sources of information and how they trust the information from these various sources, we think it will be possible to identify the key influencers who should be engaged in order to boost knowledge of PMTCT and, subsequently, adherence amongst HIV-positive mothers.

## Materials and methods

### Study design, setting, and population

With a cross-sectional study design, we enrolled 524 mothers attending PMTCT clinics in the seven districts of the Kilimanjaro region, northern Tanzania. The clinics were chosen based on statistics that were available from the district medical officers of the respective districts. The five clinics with the highest PMTCT client numbers in each district were selected. Two additional clinics from referral-level facilities in the region were also included, as they served as referral centers and mentors for the other clinics in the region. This brought the total number of facilities (PMTCT clinics) that participated in the study to 37. The facilities where these clinics were conducted ranged from dispensaries (facilities providing only out-patient services), to health centers (facilities with admission capacity of less than 20 patients) and hospitals. The clinics where the study was conducted provided antenatal and postnatal PMTCT services, which included HIV testing for both mother and child, provision of antiretroviral therapy, provision of prophylaxis for HIV-exposed children, and counseling on HIV prevention and treatment. Counseling on breastfeeding and infant feeding is also done. There is, however, no standardized counseling schedule across the clinics, as the mothers are booked at different times and have different demands. At the time of the study, none of the clinics were using any checklist that would have allowed for counter-checking of what the individual mothers had covered during their counseling sessions. As counseling is a continuous process, mothers are meant to receive counseling at every visit, but we could not confirm to what extent this was done. We thus relied on the mothers for information on what they were taught during their counseling sessions. Clients for the clinics are scheduled to attend once a month for their follow-up and provision of PMTCT-related services. Our team of research assistants visited each of the facilities in the study on their respective clinic days for a period of one month in order to capture as many clients as possible during the monthly clinic cycle. All mothers who attended the PMTCT clinics during the period of the study were invited and recruited into the study.

The mothers recruited into our study were postnatal and had children younger than two years of age receiving care in the respective PMTCT clinics. This group was chosen as we felt they would have gone through the program since their pregnancy, and we expected that this experience with the program would put them in a better position to describe the situation in the PMTCT program. The youngest child they had currently enrolled in the PMTCT program was regarded as the reference child in this study. Recruitment was done during their routine PMTCT clinic attendance.

### Ethics

The Kilimanjaro Christian Medical University College Research Ethics Committee provided the ethical clearance for this study, issuing certificate number 2434. We also sought and received permission from the regional medical officer for the Kilimanjaro region and the district medical officers of the respective districts where the study was conducted. The heads of the facilities and clinics that participated also permitted the study. Mothers were informed of the study by the research assistant, and those who agreed were asked to sign a written consent. To protect patient privacy, all interviews were conducted in consultation rooms at the respective clinics.

### Data collection

Data were collected through a questionnaire we developed and piloted on mothers in clinics not participating in the study. The questionnaire collected information on sociodemographic characteristics, antenatal history, PMTCT history, clinic attendance, PMTCT knowledge, and interactions with peers and healthcare workers (HCWs). The tool was developed first in English and then translated to Kiswahili (the national language of Tanzania) before being back-translated to ensure consistency. The final Kiswahili version of the tool was digitized on Survey-CTO^TM^ and administered by trained research assistants using password-encrypted tablets.

Data collection was carried out between the 24th of September 2019 and the 26th of February 2020. Recruitment was done by the research assistants from the client waiting areas in the PMTCT clinics. A written consent form was given to the potential participant, and the recruiter would offer any clarifications the mother needed. Those who accepted were asked to sign the consent and were given the option of having the interviews before or after their clinic visits.

The research assistants who conducted the interviews were medical students trained by the principal investigator. HCWs were neither present during the interviews nor privy to the participants’ responses. The interviews were conducted within the health facilities but out of earshot of workers or other patients. The intention of the research was reiterated to the participants during the interviews, including the assurance of anonymity. To further prevent any undesired repercussions from coming to the respondents, no information from the individual facilities was shared with the facilities. The facilities and health administration received only the aggregate report that combined the responses from all 37 facilities.

### Measures

In this study, we asked mothers whether they discussed PMTCT with other patients/ their peers or not (Yes/No response). Among mothers who answered ‘Yes’, we further asked what they discussed during clinics (breastfeeding duration, mixed feeding, and when to stop breastfeeding) and whom they trusted between their fellow mothers and HCWs. Among mothers with two or more pregnancies while HIV positive, we asked them whether they received breastfeeding advice during pregnancy with a reference child (i.e., the youngest child they had currently enrolled in the PMTCT program), and if so, whether the advice changed much between pregnancies, and for those who answered ‘Yes’, which advice they trusted (previous or current).

Other explanatory variables included the mother’s age (≤24, >24 years), the highest education level (primary or below, post-primary), marital status (single, married, cohabiting, divorced/separated/widowed), years since HIV diagnosis (≤5, >5), disclosed HIV status to partner (Yes/No), number of antenatal care (ANC) visits (<4, ≥4), and number of pregnancies while HIV positive (1, >1). We also asked mothers whether they received support from HCWs to breastfeed during ANC visits and after delivery (Yes/No), and whether they received any advice on ART in the most recent pregnancy and before discharge (Yes/No).

### Data analysis

STATA® version 15.1 was employed to perform the data analysis. Frequencies and percentages summarized the categorical data. The Chi-squared test compared the proportions of mothers discussing PMTCT with their peers (yes/no) by background characteristics. Likewise, descriptive statistics also evaluated the breastfeeding advice women received during pregnancy, specifically, whether they received advice during pregnancy with the reference child (yes/no), whether the advice changed much between pregnancies among those who received it (yes/no), and if so, which advice they trusted the most (previous vs. current). In addition, frequencies and percentages of women discussing PMTCT with their peers, the advice they trust (i.e., between their peers and HCWs, considering also the previous and current advice), and the topics discussed among their peers are also summarized. We also assessed differences in breastfeeding practices by discussing PMTCT with fellow patients/peers relative to HCWs using the Chi-squared test and found no statistically significant associations. All statistical tests were two-sided at a 5% significance level.

## Results

A total of 524 mothers participated in the study, the minority (17.7%) being youth (less than 24 years of age). Of these mothers, 289 (55%) reported having discussions with other mothers regarding PMTCT when they were in the clinic. This was in addition to the discussions with the HCWs. The proportion of women discussing PMTCT with other mothers increased by the number of pregnancies a woman ever had. The highest proportion was among mothers with four or more pregnancies (63.1%), followed by those who reported 2 to 3 pregnancies (53.2%). Those who reported having received antenatal counseling on breastfeeding were also more likely to engage in discussions with other mothers. The health facility level at which the mother was receiving care also influenced whether they engaged in discussions, with a larger proportion of those in health centers (small hospitals with an admission capacity of less than 20 patients) participating in PMTCT discussions. The strongest association seems to be with whether the mother was counseled on breastfeeding during antenatal care (ANC), where nearly sixty percent (58.4%) of these women discussed PMTCT with fellow mothers (Table 1).

**Table 1:** Proportion of women discussing PMTCT with other patients by participant characteristics.

Characteristics	Total	Discussing PMTCT with other patients		P-value
		No	Yes	
**Total**	524	235 (44.8%)	289 (55.2%)	
**Mothers age category**				0.53
**24 and below (Youth)**	93	39 (41.9)	54 (58.1)	
**Above 24**	431	196 (45.5)	235 (54.5)	
**Mother’s highest education level**				0.51
**Primary and below**	349	153 (43.8)	196 (56.2)	
**Post-primary**	175	82 (46.9)	93 (53.1)	
**Marital status**				0.52
**Single**	110	46 (41.8)	64 (58.2)	
**Married**	237	106 (44.7)	131 (55.3)	
**Cohabiting**	100	51 (51.0)	49 (49.0)	
**Divorced/Sep/Wid**	77	32 (41.6)	45 (58.4)	
**The approximate income per month**				0.12
**None**	25	10 (40.0)	15 (60.0)	
**<85,000 Tshs**	237	118 (49.8)	119 (50.2)	
**85,000+ Tshs**	262	107 (40.8)	155 (59.2)	
**Years since diagnosis**				0.60
**5 years or less**	374	165 (44.1)	209 (55.9)	
**More than 5 years**	150	70 (46.7)	80 (53.3)	
**Years on ART**				0.73
**1 to 2**	281	128 (45.6)	153 (54.4)	
**>2**	243	107 (44.0)	136 (56.0)	
**Mother disclosed HIV status to her partner**				0.49
**No**	234	101 (43.2)	133 (56.8)	
**Yes**	290	134 (46.2)	156 (53.8)	
**Number of pregnancies woman ever had**				0.02
**1**	100	54 (54.0)	46 (46.0)	
**2 to 3**	248	116 (46.8)	132 (53.2)	
**4+**	176	65 (36.9)	111 (63.1)	
**Level of the facility**				0.01
**Dispensary**	62	31 (50.0)	31 (50.0)	
**Health center**	225	84 (37.3)	141 (62.7)	
**Hospital**	237	120 (50.6)	117 (49.4)	
Number of ANC visits prior to delivery*				0.06
**Less than 4**	65	37 (56.9)	28 (43.2)	
**4 or more**	453	194 (42.8)	259 (57.2)	
**Pregnancies while HIV positive**				0.21
**1 pregnancy**	344	161 (46.8)	183 (53.2)	
**>1**	180	74 (41.1)	106 (58.9)	
**Received breastfeeding support from HCW after delivery**				0.12
**No**	455	210 (46.2)	245 (53.8)	
**Yes**	69	25 (36.2)	44 (63.8)	
**Receive breastfeeding advice from a HCW during antenatal visits**				0.001
**No**	71	45 (63.4)	26 (36.6)	
**Yes**	447	186 (41.6)	261 (58.4)	
Receive any advice from a HCW on antiretroviral treatment (ART) during pregnancy*				0.96
**No**	40	18 (45.0)	22 (55.0)	
**Yes**	478	213 (44.6)	265 (55.4)	
**Counseled on use of ART before discharge**				0.94
**No**	124	56 (45.2)	68 (54.8)	
**Yes**	400	179 (44.8)	221 (55.3)	

* Frequencies do not tally with the total (N = 524) because six (6) women did not attend antenatal care during the current pregnancy.

**Fig 1:**
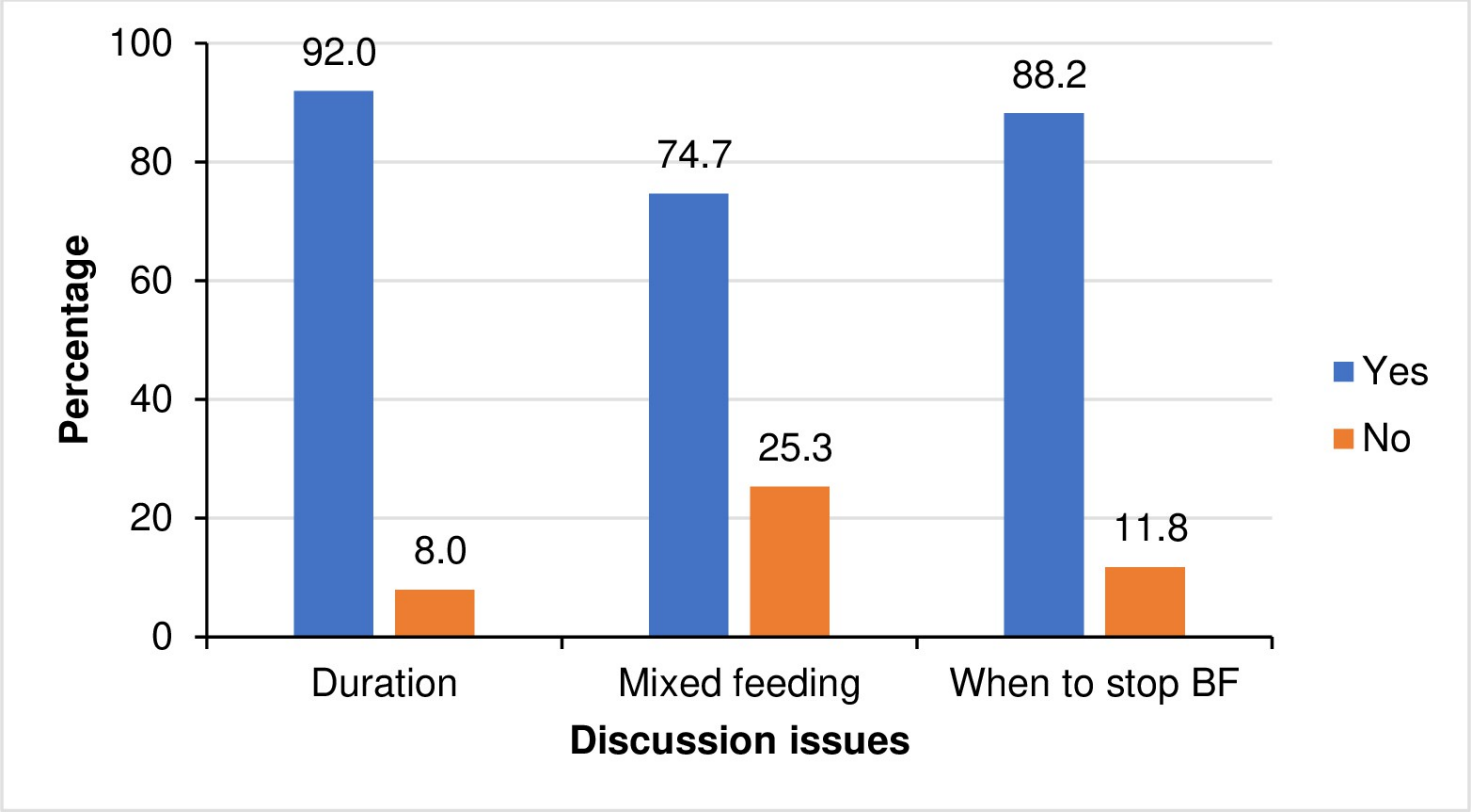
What women discuss with each other during clinics (N = 289).

The most common topics mothers discussed with each other included the duration of breastfeeding (reported by 92% of mothers), exclusive breastfeeding (reported by 74.7% of mothers), and when to stop breastfeeding (reported by 88.2% of mothers) (Fig 1).

**Fig 2:**
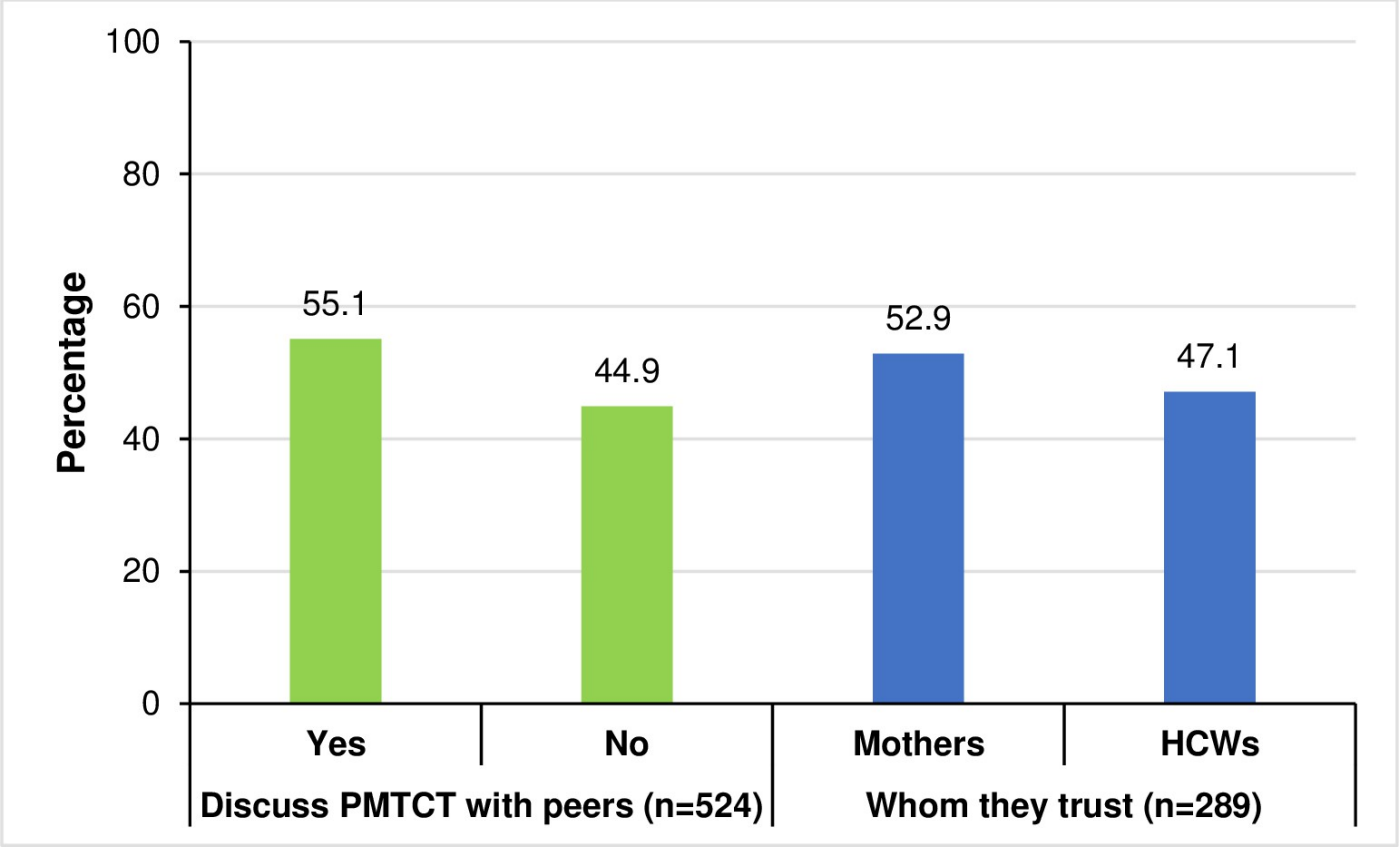
Proportion of women discussing PMTCT with other patients and which advice they trust.

Of the 524 mothers interviewed, 55.1% (289) said they discuss various issues related to PMTCT with their fellow patients when they come to the clinic. There were no statistically significant differences between whom the women discussed with (fellow patients vs. HCWs) by breastfeeding practices (results not presented), i.e., breastfeeding initiation, colostrum feeding, exclusive breastfeeding, breastfeeding up to 12 months, mixed feeding, and PMTCT knowledge. Of these 289 mothers, 153 (52.9%) went on to say that they trusted the advice they got from the discussions with fellow patients/peers more than that they received from the HCWs (Fig 2).

Of the mothers we interviewed, 178 had, in addition to the reference child, delivered at least one other child while HIV positive. Ninety-three (55%) of the 178 said the advice regarding breastfeeding in the reference pregnancy differed notably from what they had been advised during their previous pregnancies. Of the 90 (3 excluded as they reported not trusting any of the recommendations) women who reported to have noticed significant changes in breastfeeding recommendations in between their pregnancies, 33 (36.7%) said they trusted the recommendations they had previously more than what is currently being recommended (Table 2).

**Table 2:** Breastfeeding advice during pregnancy (N = 524).

Variable	Frequency	Percentage
**Number of pregnancies while HIV positive**		
**1**	346	66.0
**2+**	178	34.0
**Received breastfeeding advice during pregnancy with reference child (n = 178)**		
**No**	9	5.1
**Yes**	169	94.9
**Advice changed much between pregnancies (n = 169)**		
**No**	76	45.0
**Yes**	93	55.0
Which advice do you trust? (n = 90)*		
**Previous**	33	36.7
**Current**	57	63.3

* Excluded three individuals who reported they don’t trust any advice.

**Fig 3:**
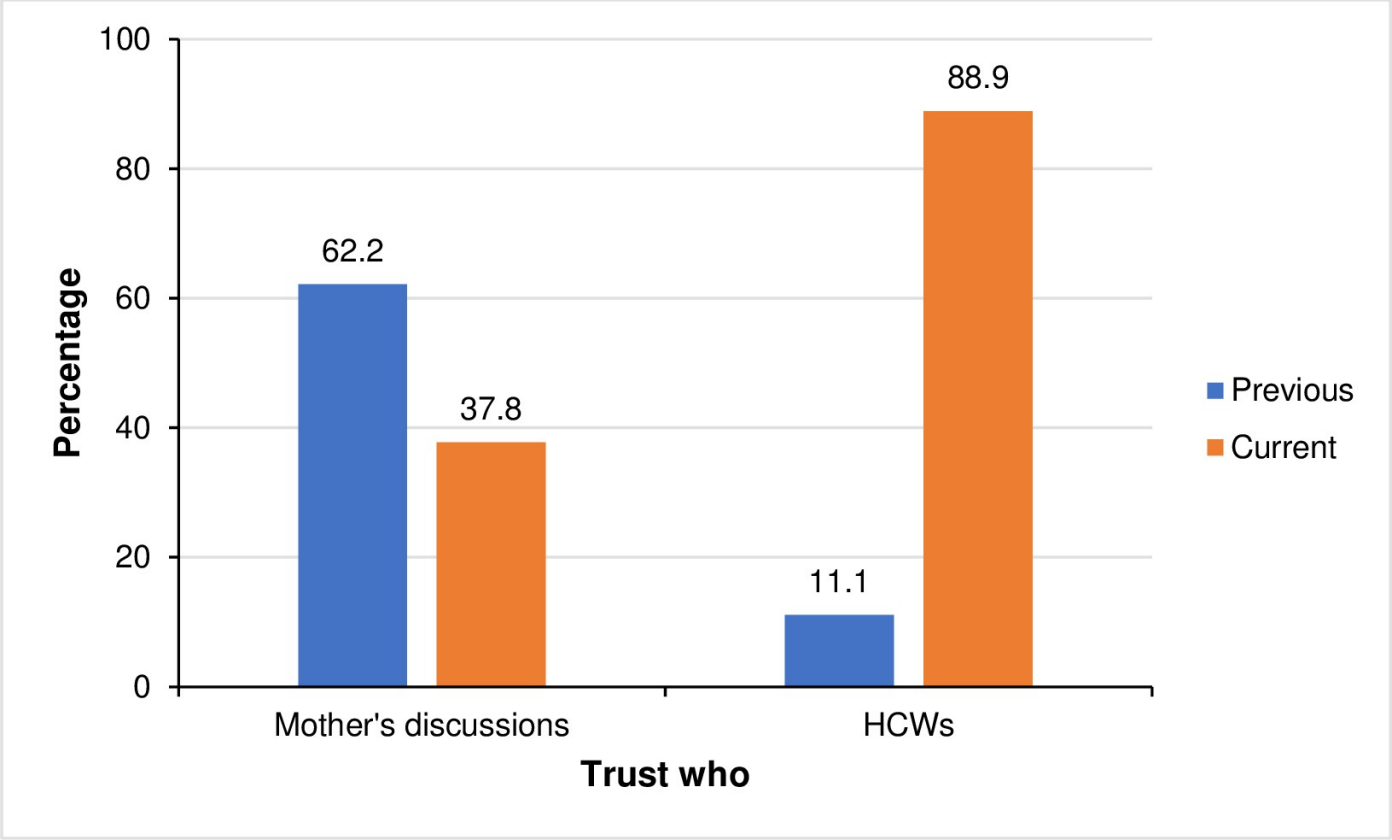
Trust in current vs. previous recommendations vs. trust in HCWs and mothers’ (peer) discussions (n = 90*). * Mothers with two or more pregnancies while HIV positive and noticed changes in advice between pregnancies.

When looking further at the women who reported having noticed changes in the PMTCT recommendations, we found that those who trusted the previous PMTCT recommendations were also more likely to trust the discussions they had with other mothers over what the healthcare providers were telling them currently (Fig 3).

## Discussion

About 34% of the mothers in our study had delivered at least one other child prior to the reference child while HIV-positive, giving them previous encounters with the PMTCT program. The majority that received counseling on breastfeeding had noticed a change in the recommendations between what they were told during a previous pregnancy and what they were told to do during the reference pregnancy. Thirty-seven percent reported trusting the recommendations they had been given previously over the advice that had been given in the reference pregnancy. More than half of all mothers in this study reported discussing elements of PMTCT with other mothers, and of those who did, about 53% said they trusted the peer discussions more than the advice of the healthcare workers. Those who discussed with other mothers were more likely to trust the older breastfeeding recommendations than the current ones.

Although the World Health Organization (WHO) emphasizes counseling regarding breastfeeding as part of PMTCT, this was not universal, with 40 out of the 518 mothers who attended antenatal care not receiving this service [14]. One study revealed that the various challenges, such as increased workload in the face of staff shortages, have led to the HCWs charged with counseling mothers on infant feeding having to compromise on the content and time spent counseling [15]. This is a problem that echoes across Sub-Saharan Africa and is made worse by the changing PMTCT recommendations regarding infant feeding [15]. Lack of breastfeeding counseling is also a problem in non-PMTCT clinics, with one community-based study in Kilimanjaro region reporting that only 64.7% of mothers received any breastfeeding counseling during their antenatal clinics [16]

Mothers who received counseling also engaged in peer discussions. We speculate that since the issues mothers discussed with their peers were aligned with recommended counseling topics, the counseling they received might not have answered all their questions. Though we did not assess how satisfied they were with the counseling, studies from elsewhere that did found this to be the case [17].

Previous PMTCT encounters with differing messages from the current recommendations have been shown to influence adherence to the current recommendations negatively [12]. Our study affirms this, with 37% of those who noted a difference between the current recommendations and what was recommended during a previous pregnancy choosing to trust what was recommended previously. Concerns about trusting the changes to breastfeeding recommendations have been raised in other places, even before the adoption of the current recommendations [18]. When new recommendations come with a paradigm shift, extra support and measures are needed to get buy-in from all the stakeholders [19].

Discussions with other mothers have great potential to influence the beliefs and choices the mothers make [12, 20]. Capitalizing on this through peer educators has been demonstrated to positively influence adherence to breastfeeding recommendations [21–24]. Our study found that 53% of mothers who discuss with each other trust the advice from mothers more and that they were less likely to trust the current recommendations. This is cause for great concern, as a previous study has demonstrated the existence of misconceptions regarding PMTCT [4]. It is plausible to assume that mothers are misinforming each other, hence misplacing their trust in outdated recommendations. To have a positive benefit from mothers’ discussions, it is thus necessary to have mothers knowledgeable in the current recommendations, who will act as experts and lead these discussions.

The social-ecological framework of health education supports the idea that individual factors such as the mother’s knowledge and peers play a key role in successful PMTCT [24]. This can be seen as an opportunity to roll out peer educators in our PMTCT program, as trust between mothers is already strong. By implementing training programs for mothers to become peer educators who will then pass on the correct information regarding breastfeeding and PMTCT, we will address multiple arms of the framework, thus increasing the likelihood of success of the PMTCT program.

The use of peer educators is also strongly supported by the key principle of the social cognitive theory of learning put forward by Albert Bandura [25]. Peer educators will allow for dynamic and reciprocal interaction among mothers, which is in line with what Bandura describes as reciprocal determinism. This will be taking place in an environment and context in which both the educator and the learner (mothers in the PMTCT program) are comfortable with, further facilitating the learning process.

Though our study clearly shows the important influence that peers have toward the opinion of mothers in PMTCT, we are limited by not having looked deeper into why they have a powerful influence and what characteristics ideally curry favor and influence amongst the peers. We therefore see an opportunity for further research into these areas so as to see how best to choose a peer educator in this community and setting. Existing works in similar populations, including one in Dar-es-Salaam, Tanzania, have already shown peer educators to have a positive influence. However, they have also fallen short in describing who the ideal peer educator would be [26].

It would also be informative to look into the perspective of the HCWs as to why the trust of mothers is leaning towards their peers and older recommendations. Informal feedback from mothers that HCWs pick up might give important insights into how best to support peer educators.

Our study relied on the recall of the mothers regarding their experiences and information they might have picked up from multiple sources. Though this might be seen as a limitation in some context, we feel that since the questions we posed to them and their recollections pertained to what they understood from all their experiences, and it was what they were actually practicing. For us, this is a strength as it demonstrates what the mothers retain from their PMTCT encounters. We cannot however guarantee that it was their recollection of what they were told both by HCWs and peers was clear.

Although beyond the scope of our current study, another possible area to look into would be how the knowledge mothers have matches with the PMTCT recommendations for breastfeeding at the time of their previous encounter, as well as the HIV status of the children delivered during the previous encounters. This would shed light on the acceptability of the recommendations. It would also be worthwhile to further explore whether the previous recommendations where easier to follow as a factor that might have made mothers trust the previous recommendations.

## Conclusions

The influence of peers cannot be overlooked in PMTCT, and mothers who engage in discussions with others during their PMTCT clinic visits are more likely to trust what their fellow mothers tell them rather than what the HWCs tell them. Harnessing the influence mothers have on each other through peer educators may go a long way in improving adherence to PMTCT recommendations for breastfeeding and lowering mother-to-child transmission of HIV. The selection of who the ideal peer educator would be is still a subject that needs to be researched.
